# Investigating the neurochemistry of the human visual system using magnetic resonance spectroscopy

**DOI:** 10.1007/s00429-021-02273-0

**Published:** 2021-04-26

**Authors:** I. Betina Ip, Holly Bridge

**Affiliations:** Wellcome Centre for Integrative Neuroimaging, FMRIB Building, Nuffield Department of Clinical Neurosciences, University of Oxford, John Radcliffe Hospital, Oxford, OX3 9DU UK

**Keywords:** Magnetic resonance spectroscopy, Visual cortex, Cortical inhibition, Glutamate, GABA, Perception, Plasticity, Dysfunction

## Abstract

Biochemical processes underpin the structure and function of the visual cortex, yet our understanding of the fundamental neurochemistry of the visual brain is incomplete. Proton magnetic resonance spectroscopy (^1^H-MRS) is a non-invasive brain imaging tool that allows chemical quantification of living tissue by detecting minute differences in the resonant frequency of molecules. Application of MRS in the human brain in vivo has advanced our understanding of how the visual brain consumes energy to support neural function, how its neural substrates change as a result of disease or dysfunction, and how neural populations signal during perception and plasticity. The aim of this review is to provide an entry point to researchers interested in investigating the neurochemistry of the visual system using in vivo measurements. We provide a basic overview of MRS principles, and then discuss recent findings in four topics of vision science: (i) visual perception, plasticity in the (ii) healthy and (iii) dysfunctional visual system, and (iv) during visual stimulation. Taken together, evidence suggests that the neurochemistry of the visual system provides important novel insights into how we perceive the world.

## Introduction

Brain activity involves electrical and chemical signals. Electrical signals, in the form of action potentials, are used to propagate information along neurons, while chemical signals involve communication between cells through neurotransmitters. Indeed, the computational power of the brain comes from chemical signals between cells that can silence or increase action potentials across the brain. Chemical signals from inside the cell can represent other roles that are fundamental to brain function, including energy metabolism and supporting neuronal integrity.

It is established that non-invasive imaging methods such as electro-encephalograms (EEG) measure the electrical activity of neurons from the surface of the scalp, while functional magnetic resonance imaging (fMRI) uses the blood-oxygenation-level dependent (BOLD)-signal, a proxy of neural activity reflected in local blood flow. Yet, neither approach provides information about the chemical signals of the brain. Proton magnetic resonance spectroscopy (MRS) is currently the main tool to measure neurochemistry non-invasively in the living brain. It is readily accessible through standard brain scanners and provides three key advantages: (1) it is non-invasive and safe for repeated scanning—increasing utility for longitudinal clinical studies; (2) it is quantitative—the amplitude of the signal is directly proportional to the number of protons in a particular chemical structure and (3) it can be used to reproducibly measure biologically relevant chemicals from most regions in the brain, from the neocortex to the brain stem. A challenge in the application of MRS to biologically relevant molecules other than water is the low concentration of these metabolites in the brain. These are present at minute, millimolar concentrations. In comparison, at 65 M, water is at least 1:10.000 more abundant than metabolites (Posse et al. 2013). Because of this low concentration, MRS needs large voxels and long acquisition times to obtain data with a sufficient signal-to-noise ratio (SNR). The quantification of very low concentration metabolites or spatially overlapping signals is further improved by increasing MRI field strength (Tkac et al. 2009). In single-voxel MRS, typical volume sizes are around 8 cm^3^ (2 cm × 2 cm × 2 cm), 8000 times larger than the commonly used 1 mm isotropic resolution of a structural MRI scan (Blüml 2013). The temporal resolution of MRS is not currently known but it is generally agreed that to obtain sufficient SNR, spectra need to be acquired over several minutes.

## How does MR spectroscopy work?

**Fig. 1:**
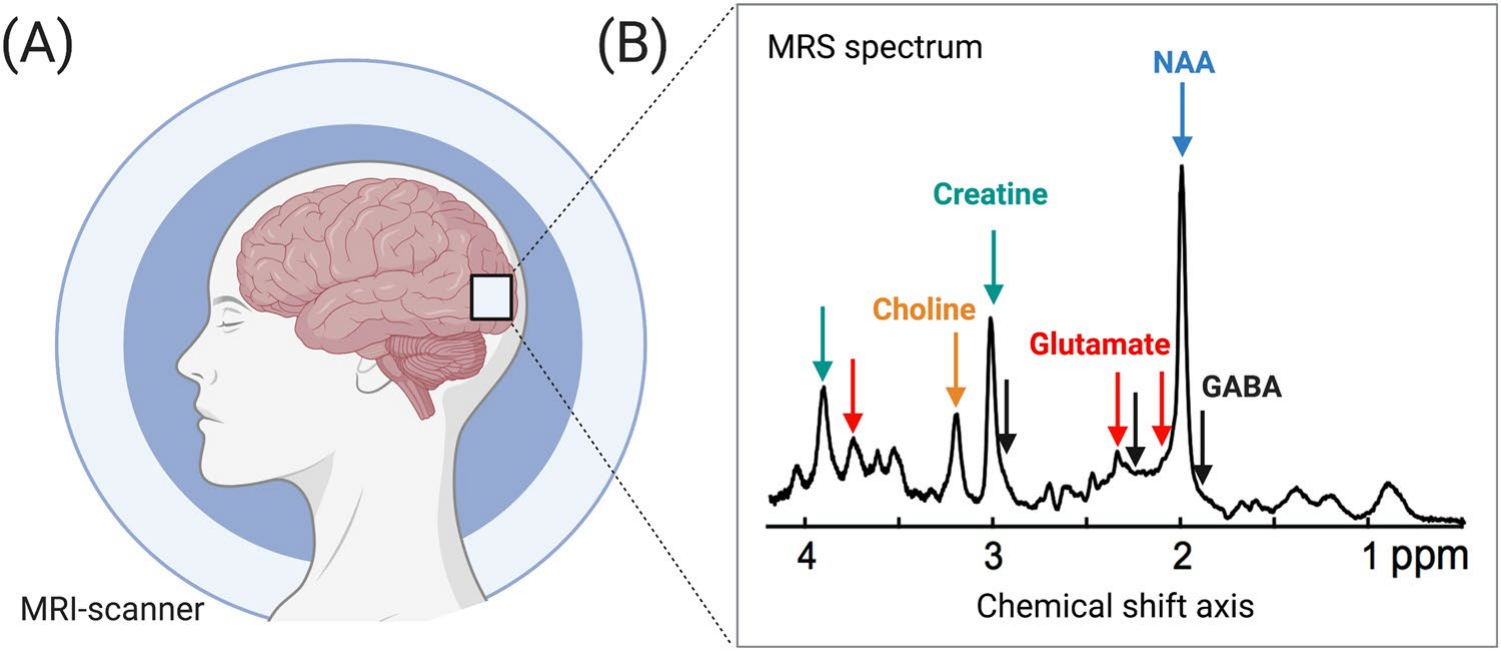
General scheme of MRS. (**a**) Metabolite signals are acquired using an MRS scan from a region of interest in the visual cortex (white box). (**b**) The MRS spectrum’s amplitude reflects the bulk signal across tissue types and cellular compartments. Metabolites are identified using prior knowledge of their chemical properties, which determine their resonant positions along the chemical shift axis. Shown from left to right are examples of metabolite peaks belonging to choline (orange), a cellular membrane marker; creatine (green), a marker of energy metabolism; excitatory neurotransmitter glutamate (red); *N*-acetyl aspartate (NAA, blue), a marker for neuronal integrity, and inhibitory neurotransmitter γ-aminobutyric acid (GABA, black). Multiple arrows of the same colour indicate that the same metabolite is composed of multiple peaks along the spectrum. In a 7T scan, the spectrum can be separated into over a dozen metabolites along the chemical shift axis. *MRS* Magnetic resonance spectroscopy, *MRI* magnetic resonance imaging, *ppm* parts per million

Nuclear Magnetic Resonance Spectroscopy (NMR) is a technique that resolves the chemical components of a sample, a method commonly associated with organic chemistry. Applied to neuroscience, as specified earlier, MRS typically refers to single voxel in vivo ^1^H-MRS, where data from a single large volume is collected. MRS takes advantage of the specific chemical structure of metabolites and its effect on resonant properties of hydrogen nuclei to quantify ‘brain chemistry’ within a defined region of cortical tissue (Fig. 1a). While standard MRI uses the most abundant molecules in the brain, water and lipids, to create detailed spatial maps of brain anatomy, MRS focuses on low concentration chemicals. Unlike MRI, MRS in its basic form produces a spectrum rather than an image of the brain. The characteristic peaks and troughs of an MRS spectrum show the strength of the signal as a function of its resonant frequency: the position along the *x*-axis identifies the frequency of the molecule in units of part per million (ppm), the *y*-axis represents the intensity  which is proportional to the number of protons resonating at that frequency of the chemical shift axis (Fig. 1b).

The single charged proton of a hydrogen nucleus gives it a magnetic momentum that renders it visible in the external magnetic field provided by an MRI scanner. When atomic nuclei with a magnetic moment are placed in a static magnetic field, they rotate at a frequency defined by the Larmor equation. However, the specific structure of the molecule, its immediate chemical environment and neighbouring chemical bonds, can further determine the exact magnitude of the static magnetic field  experienced by a hydrogen atom (Blüml 2013). A key consequence of this property is a slight shift of the resonances. Organic molecules consist of multiple hydrogen atoms, bound into specific chemical groups. Different chemical groups, such as carbonyl, benzene, oxygen, have different magnetic properties due to their surrounding electron orbits. This means that protons in identical chemical environments appear at the same position, whereas protons in different chemical environments appear at different positions. Because a molecule contains a number of protons in different chemical environments, their spectral peaks can be separated into distinct signals along the chemical shift axis. Specifically, the electrons surrounding hydrogen atoms create their own local magnetic fields that oppose, ‘shield’, the atom from the static magnetic field. Thus, atomic nuclei that are more shielded experience weaker influences from the static magnetic field than nuclei that are less shielded. The chemical shift axis in ^1^H-MRS is flanked by two extremes. At the extreme right side is the common reference signal of tetramethylsilane (TMS), this is the most shielded signal and set as a reference standard at 0 ppm. The chemical shift of a molecule is quantified as a difference in the resonant signal relative to the reference signal TMS at 0 ppm (Granger et al. 2007) and is reported in parts per million (ppm). Moving from 0 ppm to the left (or ‘down field’ from 0 ppm), signals become increasingly more susceptible to the static magnetic field, and on the extreme left is the suppressed water signal at 4.7 ppm. Most metabolites of interest appear in the narrow range of resonances between 4.7 and 0 ppm.

In summary, the known properties of chemical structures lead to their identification on the chemical shift axis. Using this knowledge, molecules of significant biological interest can be measured using MRS (Fig. 1b). These include *N*-acetyl Aspartate (NAA), a marker for neuronal integrity, creatine (Cr), an energy marker and choline (Cho), an astrocytic membrane marker. Of particular interest to neuroscience are glutamate (Glu) and *γ*-aminobutyric acid (GABA), excitatory and inhibitory neurotransmitters in the brain, respectively. Strong metabolite signals, such as NAA, Cr and Cho, can be imaged at 1.5 T. Quantification of GABA requires special MRS sequences at 3 T (Puts and Edden 2012). At 7 T, increased SNR and spectral resolution benefit both GABA and glutamate detection (Tkac et al. 2009). A major advantage of ultra-high field MRS is a more reliable quantification of glutamate, enabled by the increased spectral resolution that separates glutamate from overlapping resonances of glutamine (Tkac et al. 2001). The importance of each metabolite to brain metabolism is reviewed elsewhere (Govindaraju et al. 2000; Rae 2014) and will not be discussed in this review.

## Relating excitation and inhibition to neuronal tuning

**Fig. 2:**
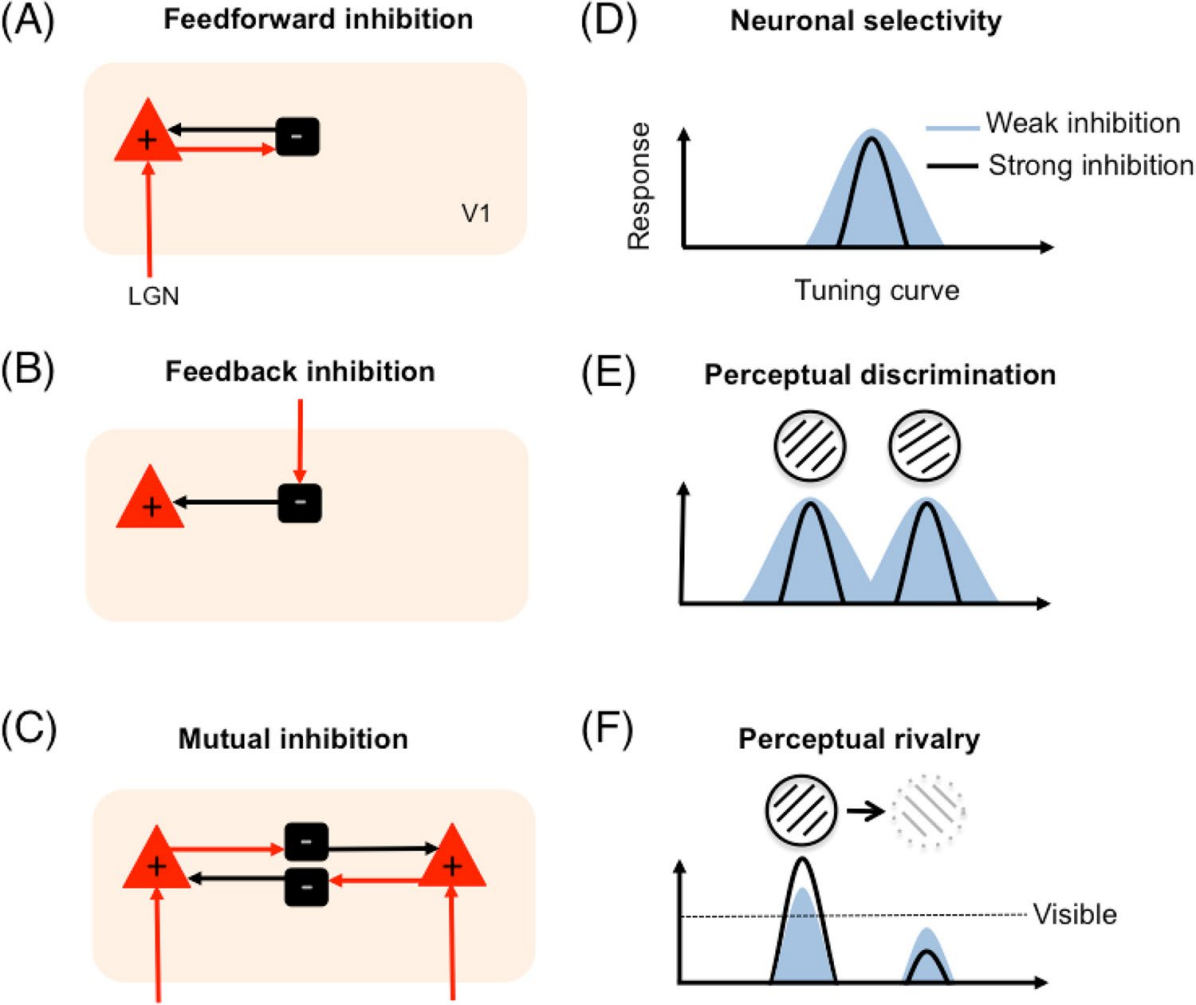
A schematic diagram of three inhibitory circuits in the primary visual cortex (V1). (**a**) A feedforward circuit involving a principal excitatory cell and an inhibitory cell. Inputs from the lateral geniculate nuclei (LGN) arrive in V1. Excitation causes recurrent inhibition onto the excitatory cell. (**b**) A feedback circuit showing an inhibitory cell receiving input from higher areas, causing inhibition onto an excitatory cell. (**c**) In mutual inhibition, cells encoding different stimuli inhibit each other during competing neural input. The more active population inhibits the rival population until a switch occurs due to habitation or noise. The interplay of excitation and inhibition can affect neuronal tuning in different ways. For example, (**d**) GABAergic inhibition can cause narrowing of the neuronal tuning curve, improving neuronal selectivity. (**e**) This could reduce the amount of overlap between similarly tuned neuronal populations and improve visual discrimination. (**f**) Mutual inhibition between populations of cells could mediate perceptual dynamics during binocular rivalry, with the visible percept suppressing the invisible percept. Stronger inhibition could translate into longer perceptual dominance durations

The cortical network is comprised of excitatory and inhibitory cells, and the spatial selectivity of visual neurons is thought to emerge from the interaction between glutamatergic excitation and GABAergic inhibition (Isaacson and Scanziani 2011). Excitatory neurons can form long-range connections to other areas with their axons, whereas inhibitory interneurons typically signal within a cortical area. In feedforward inhibition, input from the lateral geniculate nuclei (LGN) excites principal excitatory cells in the primary visual cortex (V1). These drive inhibitory cells, which in turn cause recurrent inhibition onto those principal excitatory cells (Fig. 2a). Input from higher areas arriving in the visual cortex can excite inhibitory cells and generate inhibitory feedback (Fig. 2b). In addition, mutual inhibition describes a situation where the input to one population of neurons suppresses another population responding to competing input (Fig. 2c). These dynamics are known to affect neuronal selectivity. In a neuronal tuning curve model of a highly selective neuron, the peak of the curve represents the preferred stimulus feature, the amplitude is the strength of the signal in firing rate, and the width is a measure of selectivity. Inhibition can improve the signal-to-noise ratio by narrowing the tuning curve (Fig. 2d), for example by permitting only the strongest inputs from preferred stimuli to lead to neuronal firing (Shapley et al. 2003; Isaacson and Scanziani 2011). In a perceptual task, an increase in selectivity would improve the ability to discriminate between inputs, such as between two similarly oriented gratings (Fig. 2e). In a binocular rivalry paradigm where the inputs to each eye compete for access to visual awareness (Fig. 2f), mutual inhibition between groups of neurons could facilitate input to one stimulus while suppressing input to another (Seely and Chow 2011). In summary, evidence suggests that GABAergic inhibition plays an essential role in modulating basic neuronal response properties that relate to perceptual performance. Only recently have studies started to investigate the relationship between visual performance and GABAergic inhibition using MRS.

## Linking individual metabolite concentration to visual perception

GABA can be reliably and stably measured in the brain using MRS (Puts and Edden 2012; Evans et al. 2010; Greenhouse et al. 2016), where it is present at very low concentrations (~ 1–2 micromol/g). The GABA signal is composed of three methylene groups that resonate at 1.89, 2.28 and 3.01 ppm (Govindaraju et al. 2000). These overlap with resonances of other metabolites with much stronger signals, especially with the Cr peak at 3.0 ppm, which makes their detection challenging (Mullins et al. 2014). Although GABA can be detected with several acquisition schemes (Puts and Edden 2012), the most widely used method of GABA detection is performed at 3 T using a spectral edited technique called MEGA-PRESS (‘MEscher-GArwood Point RESolved Spectroscopy’) (Mescher et al. 1998; Mullins et al. 2014). Spectral editing of GABA works on the principle that an editing pulse applied at 1.9 ppm can change the signal of the GABA peak at 3 ppm due to interactions between hydrogen nuclei in the same molecule. Subtracting a spectrum with edit ‘ON’ from a spectrum with edit ‘OFF’ removes all spectral peaks apart from those affected by the editing pulse (Mullins et al. 2014). MEGA-PRESS isolates the GABA signal at 3.0 ppm and the combined signals of glutamate, glutamine and glutathione at 3.75 ppm. An important limitation of the MEGA-PRESS sequence is that the measured GABA signal at 3.0 ppm is contaminated by co-edited resonances from macromolecules (MM) (Harris et al. 2015) and homocarnosine, a dipeptide of GABA and histidine (Rothman et al. 1997). GABA measured using MEGA-PRESS, as used by the majority of studies in this review, is therefore referred to GABA+.

**Fig. 3:**
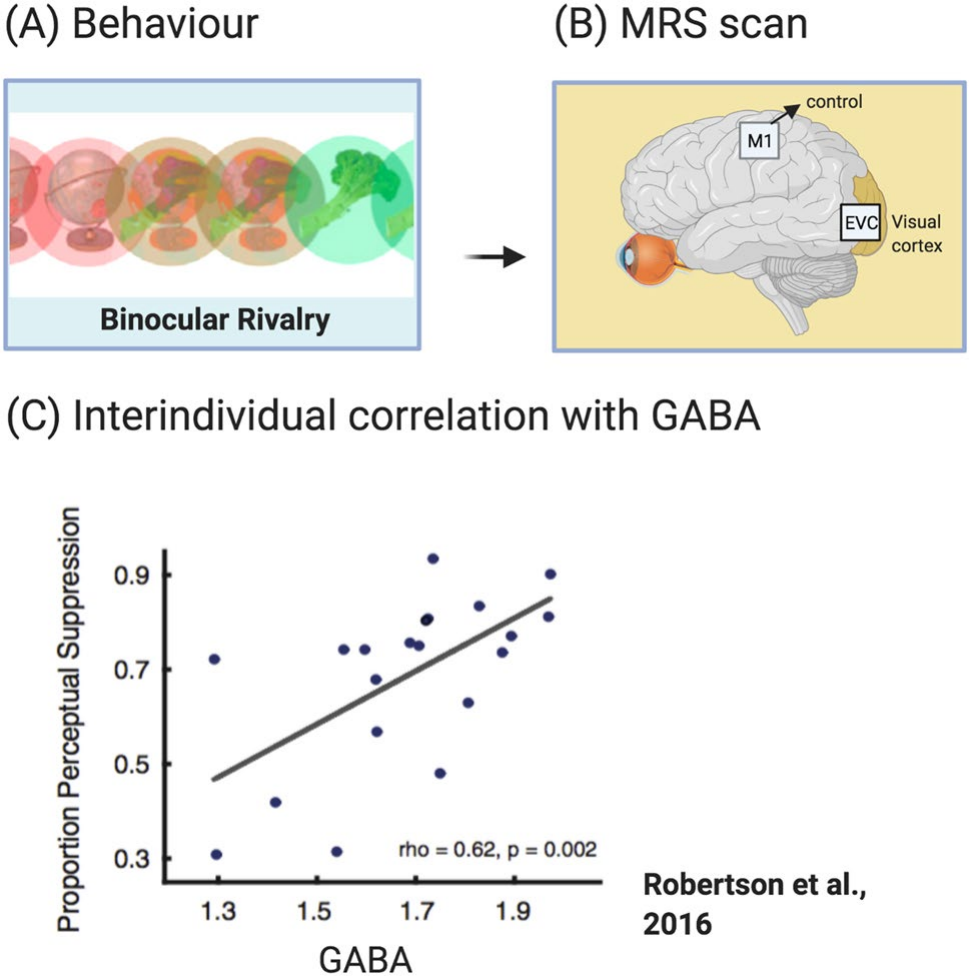
Relating interindividual variability in behavioural performance to GABAergic inhibition. The link between GABAergic inhibition and visual perception can be evaluated using MRS in combination with behavioural psychophysics. In the example by Robertson et al. (2016), (**a**) a binocular rivalry task is performed outside of the scanner. (**b**) MRS is applied in the visual cortex to quantify levels of neurochemicals, including GABA. A control voxel is placed in another location (i.e. motor cortex)  to test the regional specificity of metabolite estimates. (**c**) By correlating the behavioural with the MRS measure, the study showed that higher GABA levels related to stronger perceptual suppression. Figures reproduced with permission

Across healthy individuals, it is well-established that there is significant variability in both brain anatomy and function and performance in visual tasks. Relating interindividual variability in visual cortex GABA to perception has improved our understanding of how visual information is judged, integrated and balanced in the human brain. Most studies have measured metabolite levels in the visual cortex using ‘resting MRS’, an approach where no specific task is given to participants during data acquisition (Stanley and Raz 2018). Typically, behavioural psychophysics are acquired outside of the scanner, for example, performance on a binocular rivalry task (Fig. 3a). In addition, resting metabolite concentrations are collected in another region to provide a control for regional specificity (Fig. 3b). The measure for behavioural performance is then correlated to metabolite levels, such as in the example by Robertson et al. (2016), to evaluate the link between perception and neurochemistry.

### Visual discrimination

GABAergic inhibition in the visual cortex has been investigated in relation to visual discrimination of orientation (Edden et al. 2009; Mikkelsen et al. 2018a), motion (Schallmo et al. 2018, 2019) and visual contrast (Hammett et al. 2020). The potential link between GABAergic inhibition and visual discrimination was first pursued by Edden et al. (2009), who measured resting GABA + in the early visual cortex of volunteers who performed an orientation discrimination task outside of the scanner. Thirteen participants judged whether an oriented grating was rotated clockwise or counter-clockwise from a reference grating presented either at 0° (‘vertical’) or at 45° (‘oblique’). Both resting MRS and MEG data were collected, providing measures of neurochemistry and visually evoked gamma frequency, respectively. They found that individuals with better orientation discrimination of oblique stimuli also had higher GABA+ levels in the early visual cortex, which is consistent with the view that GABA plays a role in improving stimulus selectivity in the visual cortex (Shapley et al. 2003). Consistent with a prior study (Muthukumaraswamy et al. 2009), GABA+ also positively correlated with oscillatory signals in the gamma frequency range (30–80 Hz), a metric for the synchronised activity of cortical neurons.

Because MEGA-PRESS quantifies macromolecule (MM) contaminated GABA+, and not ‘pure’ GABA, a subsequent study (Mikkelsen et al. 2018a) set out to evaluate the contribution of pure GABA in the association between orientation discrimination and GABA+ (Edden et al. 2009). The study acquired MM-suppressed GABA in the occipital cortex and found a trend level association between MM-suppressed GABA and orientation discrimination, yet no link between GABA+ and orientation discrimination (Mikkelsen et al. 2018a). This result suggests that MM-suppressed GABA may offer greater power in detecting a relationship between cortical inhibition and perceptual performance than GABA+, and that MM can add variability to the data. The link between gamma oscillations and GABA+ has been investigated in an independent replication study (Cousijn et al. 2014). Cousijn et al., used a short-echo time MRS sequence (SPECIAL) and failed to replicate a correlation between resting GABA levels and stimulus-evoked gamma frequency in the visual cortex in a group of 50 participants. The choice of sample size and sex balance could potentially explain why the results could not be replicated, with both prior studies showing a positive association composed of moderately sized cohorts (*N* = 12 (Muthukumaraswamy et al. 2009) and *N* = 13 (Edden et al. 2009)). Participation in these two studies was limited to males to avoid possible GABA fluctuations during the menstrual cycle (Epperson et al. 2005). In contrast, Cousijn et al., (2014) involved 50 participants of both sexes. Another reason could be the difference in the MRS sequence: the SPECIAL sequence identifies all three methylene groups of GABA, while the MEGA-PRESS sequence applied by Muthukumaraswamy et al. (2009) and Edden et al. (2009) estimates GABA using the resonance at the 3 ppm position. Although the specific reason for the difference in findings remains to be determined, these studies highlight the importance of considering the limitations of the MRS sequence, alongside the size and composition of the participant cohort, to present a valid interpretation of the results.

More recently, studies have investigated the relationship between motion discrimination and neurochemistry in the human motion-sensitive complex hMT+ (Schallmo et al. 2018, 2019). Participants judged the direction of a grating drifting to the left or right at various contrast levels and sizes. The time needed to perform a threshold response was taken as the measure of motion discrimination. Across 22 observers, GABA+ concentration correlated with motion discrimination, where higher GABA+ was associated with less time needed to perform the task across all stimulus conditions. In a subsequent report, Schallmo et al. (2019), showed that higher Glx, the combined signals of glutamate and glutamine, from the same data set also correlated with better motion discrimination in hMT+. As interindividual GABA+ and Glx did not correlate with each other, the balance between excitation and inhibition within individuals is unlikely to have accounted for the observation. Taken together, these results suggest that both inhibition and excitation contribute to better motion discrimination, potentially by improving the signal-to-noise ratio and increasing neural responsiveness during perceptual tasks.

### Bistable perception

Bistable perception describes the spontaneous alternation between two possible perceptual interpretations of the same stimulus. Perceptual dynamics to bistable stimuli have been shown to relate to GABAergic inhibition. Bistable stimuli offer well-controlled paradigms to study the relationship between retinal input and the brain’s perceptual interpretation. Models of bistable perception suggest that populations of stimulus selective neurons in the visual cortex compete with each other for perceptual dominance, and that perceptual alternations are generated by mutual inhibition (Blake 1989; Seely and Chow 2011). Support for this view from studies in humans has emerged over the past 10 years from a number of different groups that have demonstrated higher occipital GABA in healthy individuals with (i) slower perceptual dynamics (van Loon et al. 2013), (ii) greater dominance duration (Lunghi et al. 2015; Pitchaimuthu et al. 2017) and (iii) stronger perceptual suppression (Robertson et al. 2016), although a lack of correlation between GABA+ and bistable motion stimuli has also been reported (Sandberg et al. 2016).

To understand cortical mechanisms during bistable dynamics, van Loon et al. (2013) combined three well-established bistable psychophysical paradigms: binocular rivalry (BR), motion-induced-blindness (MIB), and structure-from-motion (SFM), with measures of cortical inhibition from the occipital lobe using MRS. Across all paradigms, GABA+ correlated with percept duration, such that participants with higher GABA+ had longer percepts of a single interpretation and, therefore, fewer bistable switches of perception overall. No such effect was seen in a control area in the frontal cortex. To further investigate the causal influence of GABAergic inhibition on bistable perception, GABA_A_ receptor agonist lorazepam was administered to enhance GABAergic signalling. Lorazepam increased the mean percept duration for motion-induced-blindness and structure-from-motion compared to placebo, specifically through decreasing the frequency of short duration in favour of long duration percepts. The first result suggests that bistable dynamics may be regulated by a common mechanism, as dominance duration of all three paradigms correlated with GABA+. However, the pharmacological experiment suggests that there is more complexity: in motion-induced-blindness, only *invisible* durations correlated with GABA+ and in contrast, only *visible* durations increased with lorazepam. It thus appears that different perceptual metrics in the same bistable paradigm may be associated with GABA+ levels in different ways. No data were collected for binocular rivalry due to difficulty in achieving a single fused image of two stimuli that are presented separately to each eye while on lorazepam. Hence, the only bistable stimulus consistently supported by correlative and causal manipulations with GABA was structure-from-motion.

A subsequent replication analysis failed to demonstrate the association between occipital GABA+ levels and SFM percept durations using a larger cohort (*N* = 37) (Sandberg et al. 2016). Sandberg et al., argue that this may be due to a discrepancy in stimulus instructions: van Loon et al. (2013) asked participants to consciously increase alternation rates in every *even* trial run, yet the data from odd and even runs were not analysed separately. Unmodelled influences from higher areas involved in attentional control in visual areas could have facilitated cortical inhibition on neuronal populations involved in SFM perception. The null finding in Sandberg et al. may thus be related to weaker top down modulation during the behavioural performance.

### GABA and competitive balance in the binocular visual system

Normal binocular vision depends on a sensory balance in which neither eye dominates too much over the other. The balance between eyes is thought to be regulated by roughly equal reciprocal GABAergic inhibition between the anatomically segregated inputs from the left and right eye into V1. Consequently, abnormal eye dominance may be supported by unbalanced inhibition, with the stronger eye inhibiting the weaker eye’s input and causing a reduction in function. In support, direct (Sengpiel et al. 2006; Harauzov et al. 2010) and indirect (Maya Vetencourt et al. 2008) pharmacological reduction of GABAergic signalling in V1 of animals with pathological eye dominance has been shown to rescue neural function of the weaker eye. This effect was reversed by increasing GABAergic signalling (Maya Vetencourt et al. 2008). Binocular rivalry is a well-established psychophysical paradigm that gives a behavioural index of this cortical competition between eyes (Ooi and He 2020; Levelt 1965; Alais 2005). Here, rivalling stimuli presented to each eye compete for perceptual dominance, and the pattern of perceptual dynamics is thought to reflect excitation and inhibition in the brain. Recent studies have exploited the binocular rivalry paradigm to investigate the role of GABA in cortical excitation and inhibition per se (Robertson et al. 2016; van Loon et al. 2013; Mentch et al. 2019), in neural plasticity (Lunghi et al. 2015), and in ageing (Pitchaimuthu et al. 2017).

Robertson et al. (2016) used a binocular rivalry task (Fig. 3a) and MRS to investigate how visual performance and GABA + levels in control participants compared to participants with autism. Resting MRS data were acquired in the early visual cortex and in a control voxel in the motor cortex (Fig. 3b). Across neurotypical participants, higher GABA+ levels correlated with greater perceptual suppression, a measure that quantifies the proportion of time that a single eye’s percept dominates (Fig. 3c). In contrast, no such relationship was present in participants on the autistic spectrum. When the correlation analysis was applied to Glx, the combined signal of glutamate and glutamine, the authors found a positive correlation with perceptual suppression in both control and autistic participants. This study suggests a specific abnormality in inhibitory signalling in autism.

Interocular competition measured by binocular rivalry was also used by Lunghi et al. (2015) to investigate a potential link between GABAergic inhibition and brain plasticity. The dominance duration of each eye’s percept in binocular rivalry can be temporarily manipulated by patching one eye for 150 min. After removing the eye patch, the perceptual dominance of the occluded eye increases transiently (Lunghi et al. 2013). To understand whether these changes in dominance durations were related to GABA, Lunghi et al. scanned 19 participants at 7 T field strength before and after monocular deprivation. The ultra-high field strength provided a more reliable estimation of GABA through an increase in SNR and spectral resolution compared to lower field strengths (Tkac et al. 2009). Unlike prior studies, which related resting GABA directly to rivalry dominance durations (van Loon et al. 2013; Pitchaimuthu et al. 2017), the emphasis in Lunghi et al., was to quantify the impact of sensory deprivation on perception and cortical inhibition. Hence, the change in GABA before and after the intervention was the key measure of interest. Covering one eye with a patch for 150 min reliably increased the dominance duration of the deprived eye. Individuals with a stronger increase in dominance duration also exhibited a greater decrease in GABA levels after deprivation. No correlation was observed in a control voxel placed in the posterior cingulate cortex. This study demonstrated that GABAergic inhibition is important for short-term plasticity in the brain.

Visual perception, including binocular rivalry dynamics, has been shown to change with age. Pitchaimuthu et al. (2017) set out to test whether occipital GABA+ levels could be linked to differences in visual processing between younger (*N* = 20, aged 20–34) and older (*N* = 20, aged 63–78) populations. Relative to younger participants, older participants showed higher GABA+ on average. There was a significant positive correlation between occipital GABA + levels and binocular rivalry mean percept duration, however, this was only observed when both groups were combined, suggesting that the correlation may have been driven by the mean difference between groups. No correlations were observed in a control voxel placed in the pre-frontal cortex. This study demonstrates how binocular rivalry and in vivo neurochemistry can provide insights into healthy ageing.

Taken together, in vivo neurochemical studies using MRS and pharmacological interventions provide converging evidence supporting a role of cortical inhibition in regulating interocular competition in the human visual system.

### Cortical inhibition and spatial context modulation

How we see a particular object or image is determined by the spatial and temporal context in which we perceive it. The interaction with context is implemented by the receptive field structure of a neuron, which describes the spatial extent in the visual field over which a neuron is responsive. The receptive field can be divided into its classical centre and its modulatory surround. Stimuli presented to the classical receptive field are modulated by the surround, such that suppressive surrounds decrease stimulus response inside the receptive field and excitatory surrounds increase the response (Fitzpatrick 2000). Surround suppression is thought to be GABAergic and has been found at multiple stages in the visual hierarchy (Angelucci et al. 2017). To indirectly investigate surround interactions in humans, studies have taken advantage of a perceptual paradigm where the specific spatial context can cause paradoxical changes in threshold performance (Tadin et al. 2003). For example, observers need longer durations to discriminate motion in large high contrast stimuli compared to small stimuli (Tadin et al. 2003); a dynamic potentially related to surround suppression (Tadin et al. 2011). Larger stimuli could activate the inhibitory surround, thereby decreasing the response to information in the centre. Hence, a key prediction is that observers with higher GABA levels should require more information, i.e. more time, to judge a large stimulus compared to a small stimulus. A number of recent studies aimed to test this hypothesis in the human visual cortex by measuring GABA from the early visual cortex or extra-striate regions using MRS. Together, these have presented mixed evidence supporting a role of neurochemistry in surround suppression. While some studies reported a link between GABA+  and orientation (Yoon et al. 2010), contrast (Cook et al. 2016) and motion-specific (Pitchaimuthu et al. 2017) surround suppression, two recent studies have not supported a relationship (Schallmo et al. 2018, 2020).

Schallmo et al. (2018) applied multiple complementary methods, including multimodal neuroimaging, computational modelling, and pharmacology to evaluate whether higher GABA+ led to stronger surround suppression. This combination of approaches provided both a rigorous correlative and causative test of the involvement of GABA in spatial context-dependent motion perception. In addition to the early visual cortex, data were also acquired from motion sensitive area hMT+. They found no evidence for a direct relationship between surround suppression with GABA+ levels in either early visual cortex or hMT+. Furthermore, if GABA played a direct role in surround suppression, pharmacologically enhancing GABA action through GABA agonist lorazepam would increase the effect of surround suppression. Instead, lorazepam led to weaker perceptual suppression than placebo. A median split into ‘high GABA’ and ‘low GABA’ participants revealed a decrease rather than an increase in motion discrimination thresholds in people with higher GABA+, again contrary to the hypothesis. The discrepancy with previous reports (Yoon et al. 2010; Cook et al. 2016; Pitchaimuthu et al. 2017) could be due to differences in the quantification of the surround suppression effect or could reflect differences in the role of cortical inhibition in processing the specific visual stimuli used in the study. In addition, the macromolecular contamination of GABA acquired using the MEGA-PRESS sequence could have a major impact on the correlation with behaviour (Mikkelsen et al. 2018a). A recent study using a large sample size (*N* = 62) also showed no correlation between surround suppression and GABA+ (Schallmo et al. 2020). This lends further support to the view that the involvement of cortical inhibition in spatial context effects is likely to be more complex than previously thought.

## The role of GABA in visual perceptual learning

Visual plasticity is greatest in early childhood when the acquisition and optimisation of visual functions is effortless. In contrast, adults can learn new visual tasks but require a significant amount of repetition and effort (Bavelier et al. 2010). Learning new skills requires plastic changes in the brain, which are thought to be facilitated by cortical disinhibition (Letzkus et al. 2015; Barron 2020). In humans, these modulations were first demonstrated in the motor cortex (Floyer-Lea et al. 2006), and subsequently in the occipital lobe (Lunghi et al. 2015; Frangou et al. 2018, 2019). Specifically, early visual cortex disinhibition has been observed during a form of visual plasticity following patching of one eye for a short period (Lunghi et al. 2015), as well as during visual perceptual learning (Frangou et al. 2018, 2019). However, not all learning is equal, and studies of the visual cortex have demonstrated that it matters both what you learn and how long  you learn for.

Frangou et al. (2019) set out to measure GABA at ultra-high field (7 T) during learning of two visual tasks, each associated with different inhibitory processes: extracting signal-from-noise and perceptual discrimination. The authors alternately acquired neurochemicals from the occipito-temporal cortex (OTC) and the posterior parietal cortex (PPC). These regions were chosen based on their role in visual processing: OTC subserves sensory processing while PPC has been linked to decision making. Half of the observers undertook the signal-from-noise task, and half undertook the perceptual discrimination task. GABA was acquired at four time points while participants gradually improved at the task inside the scanner. This provided a temporal profile of GABA during learning, and at two different levels of the perceptual decision-making pathway. The key finding was that the direction of GABA change in OTC depended on the task, whereas PPC GABA increased in both. Specifically, GABA decreased in OTC for the signal-from-noise task, indicating that disinhibition could facilitate the extraction of feature from noise, potentially through an amplification of neuronal gain. In contrast, GABA increased during learning of perceptual discrimination, potentially representing greater suppression of task-irrelevant information through top-down attention. However, no changes were observed in glutamate signals. Together, these results show that inhibitory signals in the visual cortex vary depending on *what* the human visual system is learning while parietal cortex GABA modulates information irrespective of the task. In terms of experimental design, the study is a demonstration of how different, well-matched, psychophysical paradigms can be used to dissociate how GABAergic inhibition contributes to different perceptual mechanisms.

Shibata et al. (2017) sought to understand how learning a task beyond asymptotic performance (‘overlearning’) affects visual plasticity in the early visual cortex, and whether cortical inhibition plays a role in the perceptual effects following overlearning. The authors investigated the possibility that the amount of learning in a visual perceptual task determines whether it can be disrupted by subsequent learning of another new task (Robertson et al. 2004). Participants learned to detect the orientation of a stimulus while the noise level was increased to keep the task difficult. One group performed the task until they reached asymptotic performance, while the other continued learning for an additional 20 mins after reaching asymptotic performance. This latter ‘overlearning group' maintained performance in the initial orientation task when asked to learn a second task. In contrast, learning a second task led to a loss of performance in the initial orientation task for the former group, who stopped training as soon as they reached asymptotic performance. Shibata et al., measured how these changes related to visual cortex neurochemicals before and after learning. When comparing MRS measures before and after learning,  both glutamate and GABA+ increased in the ‘no overlearning' group, but glutamate increased more than GABA+. However, in the ‘overlearning’ group, there was a strong increase in GABA+ above pre-training levels (~15%), while glutamate remained at baseline. The results suggest that cortical excitation dominates during a typical memory stabilisation period, as represented in the ‘no overlearning group’, and that this stage can be further stabilised by overlearning which is associated with an increase in cortical inhibition.

Together, these two studies of the healthy visual system indicate a clear involvement of neurochemicals in the acquisition of novel visual skills, such as orientation detection and feature detection. While Frangou et al. (2019) stressed the importance of *what* is learnt, Shibata et al. (2017) demonstrated that *how much* it is learnt is also vital. Both studies take advantage of rigorous paradigms from visual psychophysics and innovative MRS paradigms to show how the topic of ‘plasticity’ can be further unpacked to provide information about the mechanisms of learning. Insights into the normal adult brain thus set the stage to widen the investigation into dysfunction. The next section will discuss recent insights  into the neurochemistry of neuro-ophthalmological disorders.

## Glutamate and GABA associated with visual dysfunction

Since the neurochemistry of the occipital lobe, and particularly V1, can be linked to visual plasticity in the healthy brain, this raises the question of changes that might occur as a result of visual dysfunction. The most extreme version of visual dysfunction is the total absence of light perception which can be present from birth (congenital blindness) or acquired (late blindness). In the case of congenital blindness, since the visual system has received no (or very little) light stimulation, there is significant cross-modal plasticity such that ‘visual’ areas instead respond to auditory (Coullon et al. 2015b; Watkins et al. 2013; Huber et al. 2019), language (Bedny et al. 2011, 2012; Lane et al. 2015; Watkins et al. 2012; Amedi et al. 2003), tactile (Chebat et al. 2007) and cognitive stimulation (Bedny 2017). The effect that such reorganisation might have on the underlying neurochemistry is unclear, but increases in cortical thickness (Park et al. 2009; Bridge et al. 2009) most notably in V1 are also likely to impact upon the neurochemical signature. Indeed, two studies with a small sample size that used MRS to investigate V1 in congenitally blind populations (Weaver et al. 2013; Coullon et al. 2015a) both found significant increases in creatine, choline and myo-Inositol. Coullon et al., whose study was performed in participants who had anophthalmia (absence of functional eyes), suggest that the combination of the thicker cortex and increased choline and myo-Inositol resembles immature cortex, likely reflecting the lack of visual input. In contrast, the two studies did not find consistent changes in GABA+ or glutamate, with Weaver et al. finding a decrease in GABA+, but no change in glutamate, while Coullon et al. found an increase in glutamate. A study using a macaque model of congenital blindness additionally indicated an increase in choline, but the use of only 2 animals with blindness and 2 sighted controls makes any firm conclusions difficult to interpret (Wu et al. 2013).

While congenitally blind individuals show considerable changes in neurochemistry in the early visual cortex, very few differences are evident in blindness acquired in adulthood (Bernabeu et al. 2009). The only clear change was an increase in myo-Inositol, which the authors suggest relates to an increase in glial cells, and may reflect changes resulting from a loss of input. While this study is relatively old, and MRS methodology has advanced considerably, it is also the case that the functional changes in late blindness are significantly less than in congenital blindness (Burton and McLaren 2006; Burton et al. 2002), and structural changes predominantly reflect degeneration of the occipital lobes (Reislev et al. 2016).

Neurochemical changes in blindness are likely to reflect, at least in part, the widespread structural and functional differences in the occipital lobe but there are other conditions that could be directly linked to abnormal neurochemistry. In particular, it has been proposed that migraine, particularly with visual aura or visual triggering may result from abnormal excitability in the visual brain. Early MRS investigations of the visual cortex in migraine quantified abundant metabolites such as lactate and NAA. More recent studies, however, have aimed to determine whether imbalances of GABA and glutamate may underlie abnormal visual perception. Indeed, in a study of migraine with and without aura, Bigal et al. (2008) found that GABA levels were lower in patients who had experienced severe headaches in the previous month. The sample size employed in the study was not sufficient to distinguish between migraine with and without aura. A more targeted study used adult females who had migraine with aura that was visually triggered (Bridge et al. 2015). While also using a small number of participants, this study found a significant decrease in GABA in patients compared to age- and sex-matched control participants. Moreover, there was a significant positive correlation in patients between resting glutamate concentration and visual stimulation evoked fMRI activity levels in V1. This combination of results suggests abnormal neurochemistry in the visual cortex of patients with visually-triggered migraine. In contrast, a recent study did not find any change in GABA in patients with migraine, but in this case patients were only mildly affected by migraine (Staermose et al. 2019). Thus, the extent to which neurochemical imbalance may contribute to migraine, and particularly the visual involvement, remains to be determined.

Given the role of neurochemicals in plasticity and learning discussed in the previous section, changes that occur in degenerative retinal diseases have the potential to restrict or increase the effectiveness of gene therapy or stem cell therapy performed in the retina. Any intervention that may lead to increased efficacy of treatment would be beneficial. Investigations into the effects of retinal degeneration on visual cortex neurochemistry are ongoing.

## The neurochemical basis of visual activity

**Fig. 4:**
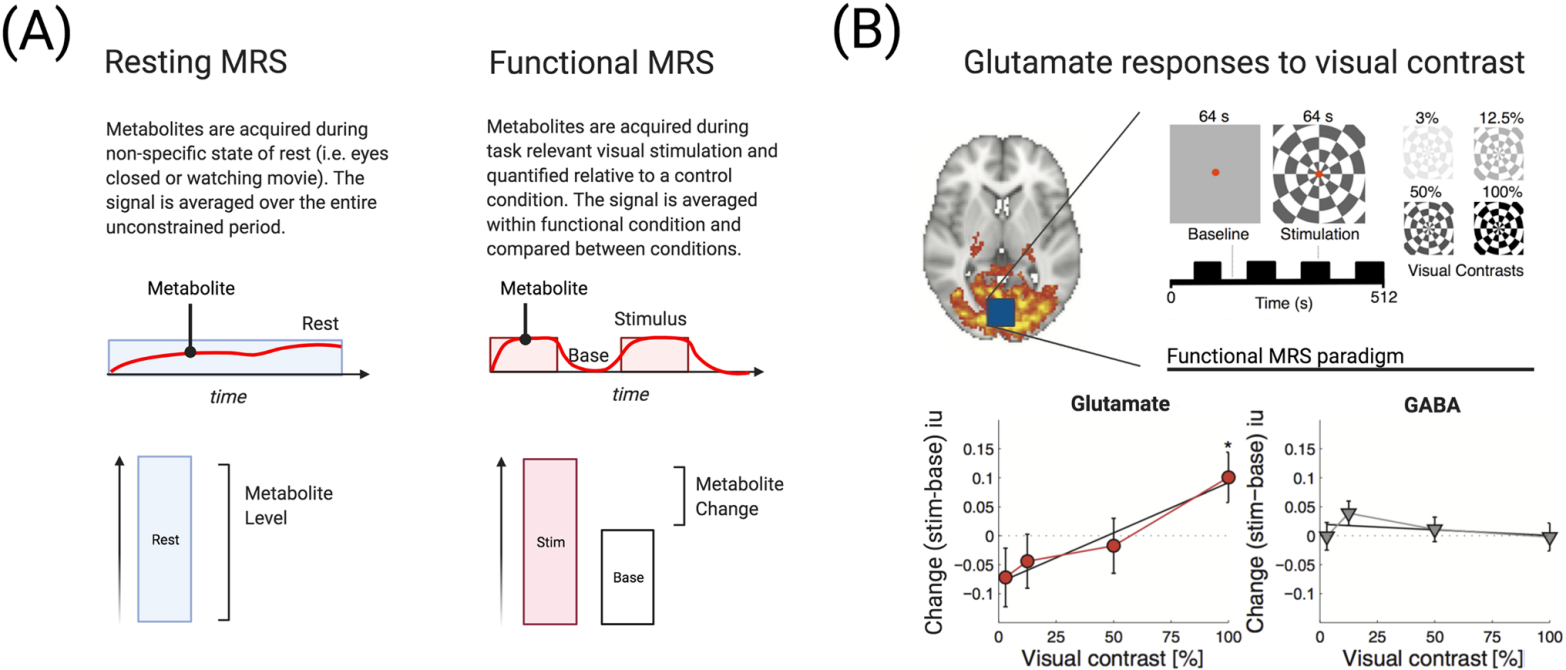
(**a**, left panel) Resting MRS is a common approach used to measure neurochemistry in the visual cortex. During resting MRS, participants watch a movie or have their eyes closed. Resting MRS taps into baseline levels of metabolites. Another approach is to use functional MRS (**a**, right panel). In fMRS, participants view task-relevant stimuli during data acquisition. (**b**) Using fMRS, Ip et al. (2019) have shown that occipital cortex glutamate increased with visual contrast whereas GABA did not (Ip et al. 2019, reproduced under author rights)

Stimulus-dependent changes are widely used in neurophysiology and neuroimaging to investigate perceptual processing, yet the majority of MRS studies on the visual system used resting MRS. A key limitation of resting MRS is that little control is exerted over sensory input or behaviour (for recent reviews, see Stanley and Raz 2018; Mullins 2018). Metabolites are hence not associated with a task-relevant perceptual state and are left to vary with little experimental constraint (Fig. 4a, right). Participants could be viewing a movie while MRS spectra are collected in the visual cortex to maintain attention and compliance. For the analysis, spectra are then averaged across the entire acquisition period to represent a ‘baseline’ measure of neurochemistry. In contrast, ‘functional’ MRS studies the biochemical pathways underlying functional changes during brain activation (Fig. 4a, left). This approach aims to reveal changes in metabolite levels, such as by comparing a functional condition with a controlled baseline state. fMRS studies have largely taken advantage of high and ultra-high field strength MRI scanners to measure low concentration metabolites during sensory processing.

Early fMRS studies used the visual system as a testing bed for studying brain metabolism. These have revealed increases in multiple metabolites during visual stimulation, including lactate and glucose (Prichard et al. 1991; Mangia et al. 2007). In addition, fMRS studies have consistently shown an increase in glutamate of about 2–4% in response to periods of long (Mangia et al. 2006; Bednarik et al. 2015; Kurcyus et al. 2018; Schaller et al. 2013; Lin et al. 2012) or short visual stimulation (Ip et al. 2017; Apsvalka et al. 2015). More recently, the fMRS methodology has been used to investigate visual processing per se, such as in response to chromatic or achromatic stimuli (Bednarik et al. 2018), the polarity of binocular images (Rideaux et al. 2019) and visual contrast (Ip et al. 2019).

In an attempt to reconcile the widely used blood-oxygenation-level-dependent (BOLD)-signal in functional MRI with neurochemical signals, a recent study (Fig. 4b) acquired fMRI and MRS in response to changing levels of visual contrast (Ip et al. 2019). Using a combined-fMRI-MRS sequence at 7 T that measures BOLD-fMRI and metabolites in the same time point, the study demonstrated that the BOLD-fMRI signal and glutamate monotonically rose with contrast levels (Fig. 4b, lower left panel). The lack of significant changes at lower contrast levels suggests a sensitivity threshold for glutamate detection measured with a functional paradigm.

GABA, on the other hand, did not change (Fig. 4b, lower right panel). While GABA has been shown to decrease during sustained activation in the motor cortex (Chen et al. 2017), evidence that GABA in the visual cortex is modulated by functional stimulation alone so far has been mixed (Ip et al. 2019; Rideaux et al. 2019; Mekle et al. 2017). The ability to detect changes in GABA during stimulation is most likely dependent on the experimental parameters, such as stimulation duration, stimulus intensity and task requirements. Task-driven GABA changes in the visual cortex have been more robustly linked to protocols involving a change in performance, whether this be through passive sensory deprivation (Lunghi et al. 2015) or active learning (Frangou et al. 2018, 2019; Shibata et al. 2017).

Hence, the most relevant evidence to visual neuroscience so far from fMRS supports a link between basic visual processing and glutamate, jointly involved in neurotransmission and energy production (Shen et al. 1999). The ability to measure neurochemistry during brain function brings us a step closer to understanding the excitatory and inhibitory nature of neural responses, yet many important questions remain, including how dynamic signals can be related to a specific function and cellular compartments. Glutamate, for example, is a major excitatory neurotransmitter and metabolite. Because MRS measures bulk glutamate concentrations irrespective of tissue type, it could reflect glutamate in extra-cellular or cellular locations. Glutamate could be in the cytosol, bound within vesicles or inside of astrocytes, where extra-cellular glutamate is transported to be broken down into glutamine as part of the glutamate-glutamine cycle. The origin and metabolic pathways of MRS visible glutamate or GABA is not discernible using MRS and is an ongoing topic of investigation. Some gaps between the cellular and population activity level can be addressed by investigation in the rodent model, where combined recordings of fMRI, MRS and optogenetics may provide a more detailed picture of brain activation (for recent review see Just 2020). While the study of the resting brain’s chemistry will surely remain a common approach, the development of functional MRS marks an exciting step towards the neurochemistry of perceptual awareness.

## Challenges and future developments

MRS is a rapidly changing field, and technical advances in recent years have led to the development of several approaches for pre-processing, analysis and quantification of spectral data. A drawback of rapid development is that the field requires standardisation before the broader community can apply MRS (Near et al. 2020). To fill this need, expert-led consensus reports provide recommendations on best practices, and the availability of open-source software such as FSL-MRS (Clarke et al. 2020) make the method more widely useable by providing a modular, end-to-end pipeline for MRS analysis. Adapting minimum standards in data acquisition (Wilson et al. 2019) can improve data quality, and using standardised protocols for processing and reporting of MRS methodology (Lin et al. 2021) can improve the transparency and reproducibility of MRS. An important area of improvement will be the enhanced separation of macromolecular contributions from metabolite signals. This is critical for MEGA-PRESS studies, as the GABA+ peak at 3 ppm has significant contributions from co-edited macromolecules (MM). MM contributions to GABA+ in the occipital lobe can reach up to 50%, and GABA+ does not necessarily correlate with MM suppressed ‘pure’ GABA (Harris et al. 2015; Mikkelsen et al. 2018a). Researchers interested in GABA estimates also need to be aware of potential changes in MM levels as a function of disease and age. Differences in MM levels have also been demonstrated between brain regions, and between gray and white matter (Cudalbu et al. 2020). Hence, it needs to be acknowledged that MM can impact on GABA+ measurements and the results interpreted appropriately (Bhattacharyya 2014). Recent recommendations on how to improve knowledge and estimation for MM have been published elsewhere (Cudalbu et al. 2020).

Spatial precision is one of the hallmarks of visual neuroscience, yet the spatial resolution of standard MRS (8–27 cm^3^) is very coarse to allow sufficient SNR. This means that visual areas, which can be functionally localised using fMRI, can be overlapping but not isolated by the MRS voxel. The neurochemical concentration of the target area is hence summed with concentrations of nearby regions, an issue that is common to single-voxel MRS studies. However, reducing the voxel size requires an increase in acquisition time to maintain equivalent signal. This can be problematic, not least because of an increased risk of scanner frequency drift and participant head motion that can impact on spectral quality. In general, shorter scan durations are preferred to enable multiple regions to be measured within the scanning session and to enhance participant compliance (Mikkelsen et al. 2018b). Another approach is to exploit the increased spatial resolution of MRS-imaging (MRSI). Although employing MRSI is technically challenging, it allows metabolites to be measured at increasingly higher spatial resolution (i.e. in-plane resolution 5 × 5 mm and thickness 10 mm; Steel et al. 2018), and greater coverage using simultaneous acquisition of multiple voxels. Thus, with a more suitable spatial resolution, studies may reflect measurements more specifically from V1 rather than larger regions of cortex, such as ‘early visual cortex’. Relatively little is known about the temporal properties of dynamic metabolite responses, as typically spectra need to be collected over several minutes to achieve sufficient SNR. This sets a limit on the ability to study the temporal dynamics of metabolite responses. While fMRS studies have revealed dynamic changes in metabolite levels in response to functional stimulation (Stanley and Raz 2018), clear quantitative relationships between stimulus and response are still to be described. This is an important area to clarify, as recent evidence suggests that metabolite dynamics, even in the resting visual cortex, can reflect reliable, regionally specific interactions between cortical excitation and inhibition (Rideaux 2020).  Taken together, these results point towards a complex relationship between metabolite levels during stimulation and rest that have yet to be investigated in detail.

Finally, a factor that is often overlooked is the state of rest during MRS data acquisition. Resting MRS is widely used in the literature, yet it would be difficult to assume that all resting conditions are the same. Recent studies have demonstrated that the state of the participant during resting MRS influences metabolite levels, with the type of rest impacting on mean concentration and variability from prefrontal (Lynn et al. 2018) to occipital (Kurcyus et al. 2018) cortices. This evidence suggests that controlling the state of the visual cortex during rest may be as important as controlling for the content of visual psychophysics. Yet, few studies report what exactly the participant was doing while MRS data were collected. The variability in visual state during resting MRS could be a potential contributing factor to the difficulty in replicating prior findings.

## Conclusions

In summary, rapid technical advances in recent years have allowed the quantification of multiple metabolites from the visual system with greater sensitivity and reliability than ever before, including neurotransmitters GABA and glutamate. MR spectroscopy is establishing itself as an important non-invasive brain imaging technique for basic vision science, enabling novel insights into the chemical signals underlying normal and abnormal visual function. Vision scientists are now exploiting this capacity, with findings supporting a varied and complex role for neurochemistry in the visual system from perception to plasticity. While numerous substantial challenges remain, in vivo neurochemistry has already built a crucial bridge, connecting invasive work in animals, perception and the biochemistry of human visual function.

## References

[CR1] Alais DBR (2005). Binocular rivalry.

[CR2] Amedi A, Raz N, Pianka P, Malach R, Zohary E (2003). Early 'visual' cortex activation correlates with superior verbal memory performance in the blind. Nat Neurosci.

[CR3] Angelucci A, Bijanzadeh M, Nurminen L, Federer F, Merlin S, Bressloff PC (2017). Circuits and mechanisms for surround modulation in visual cortex. Annu Rev Neurosci.

[CR4] Apsvalka D, Gadie A, Clemence M, Mullins PG (2015). Event-related dynamics of glutamate and BOLD effects measured using functional magnetic resonance spectroscopy (fMRS) at 3T in a repetition suppression paradigm. Neuroimage.

[CR5] Barron HC (2020). Neural inhibition for continual learning and memory. Curr Opin Neurobiol.

[CR6] Bavelier D, Levi DM, Li RW, Dan Y, Hensch TK (2010). Removing brakes on adult brain plasticity: from molecular to behavioral interventions. J Neurosci.

[CR7] Bednarik P, Tkac I, Giove F, DiNuzzo M, Deelchand DK, Emir UE, Eberly LE, Mangia S (2015). Neurochemical and BOLD responses during neuronal activation measured in the human visual cortex at 7 Tesla. J Cereb Blood Flow Metab.

[CR8] Bednarik P, Tkac I, Giove F, Eberly LE, Deelchand DK, Barreto FR, Mangia S (2018). Neurochemical responses to chromatic and achromatic stimuli in the human visual cortex. J Cerebr Blood F Met.

[CR9] Bedny M (2017). Evidence from blindness for a cognitively pluripotent cortex. Trends Cogn Sci.

[CR10] Bedny M, Pascual-Leone A, Dodell-Feder D, Fedorenko E, Saxe R (2011). Language processing in the occipital cortex of congenitally blind adults. Proc Natl Acad Sci U S A.

[CR11] Bedny M, Pascual-Leone A, Dravida S, Saxe R (2012). A sensitive period for language in the visual cortex: distinct patterns of plasticity in congenitally versus late blind adults. Brain Lang.

[CR12] Bernabeu A, Alfaro A, Garcia M, Fernandez E (2009). Proton magnetic resonance spectroscopy (1H-MRS) reveals the presence of elevated myo-inositol in the occipital cortex of blind subjects. Neuroimage.

[CR13] Bhattacharyya PK (2014). Macromolecule contamination in GABA editing using MEGA-PRESS should be properly accounted for. Neuroimage.

[CR14] Bigal ME, Hetherington H, Pan J, Tsang A, Grosberg B, Avdievich N, Friedman B, Lipton RB (2008). Occipital levels of GABA are related to severe headaches in migraine. Neurology.

[CR15] Blake R (1989). A neural theory of binocular rivalry. Psychol Rev.

[CR16] Blüml S, Blüml S, Panigrahy A (2013). Magnetic resonance spectroscopy: basics. MR spectroscopy of pediatric brain disorders.

[CR17] Bridge H, Cowey A, Ragge N, Watkins K (2009). Imaging studies in congenital anophthalmia reveal preservation of brain architecture in 'visual' cortex. Brain.

[CR18] Bridge H, Stagg CJ, Near J, Lau CI, Zisner A, Cader MZ (2015). Altered neurochemical coupling in the occipital cortex in migraine with visual aura. Cephalalgia.

[CR19] Burton H, McLaren DG (2006). Visual cortex activation in late-onset, Braille naive blind individuals: an fMRI study during semantic and phonological tasks with heard words. Neurosci Lett.

[CR20] Burton H, Snyder AZ, Conturo TE, Akbudak E, Ollinger JM, Raichle ME (2002). Adaptive changes in early and late blind: a fMRI study of Braille reading. J Neurophysiol.

[CR21] Chebat DR, Rainville C, Kupers R, Ptito M (2007). Tactile-'visual' acuity of the tongue in early blind individuals. NeuroReport.

[CR22] Chen C, Sigurdsson HP, Pepes SE, Auer DP, Morris PG, Morgan PS, Gowland PA, Jackson SR (2017). Activation induced changes in GABA: functional MRS at 7T with MEGA-sLASER. Neuroimage.

[CR23] Clarke WT, Stagg CJ, Jbabdi S (2020). FSL-MRS: an end-to-end spectroscopy analysis package. Magnet Reson Med.

[CR24] Cook E, Hammett ST, Larsson J (2016). GABA predicts visual intelligence. Neurosci Lett.

[CR25] Coullon GS, Emir UE, Fine I, Watkins KE, Bridge H (2015). Neurochemical changes in the pericalcarine cortex in congenital blindness attributable to bilateral anophthalmia. J Neurophysiol.

[CR26] Coullon GS, Jiang F, Fine I, Watkins KE, Bridge H (2015). Subcortical functional reorganization due to early blindness. J Neurophysiol.

[CR27] Cousijn H, Haegens S, Wallis G, Near J, Stokes MG, Harrison PJ, Nobre AC (2014). Resting GABA and glutamate concentrations do not predict visual gamma frequency or amplitude. P Natl Acad Sci USA.

[CR28] Cudalbu C, Behar KL, Bhattacharyya PK, Bogner W, Borbath T, de Graaf RA, Gruetter R, Henning A, Juchem C, Kreis R, Lee P, Lei HX, Marjanska M, Mekle R, Murali-Manohar S, Povazan M, Rackayova V, Simicic D, Slotboom J, Soher BJ, Starcuk Z, Starcukova J, Tkac I, Williams S, Wilson M, Wright AM, Xin LJ, Mlynarik V (2020). Contribution of macromolecules to brain H-1 MR spectra: experts' consensus recommendations. NMR Biomed.

[CR29] Edden RA, Muthukumaraswamy SD, Freeman TC, Singh KD (2009). Orientation discrimination performance is predicted by GABA concentration and gamma oscillation frequency in human primary visual cortex. J Neurosci.

[CR30] Epperson CN, O'Malley S, Czarkowski KA, Gueorguieva R, Jatlow P, Sanacora G, Rothman DL, Krystal JH, Mason GF (2005). Sex, GABA, and nicotine: the impact of smoking on cortical GABA levels across the menstrual cycle as measured with proton magnetic resonance spectroscopy. Biol Psychiat.

[CR31] Evans CJ, McGonigle DJ, Edden RAE (2010). Diurnal stability of gamma-aminobutyric acid concentration in visual and sensorimotor cortex. J Magn Reson Imaging.

[CR32] Fitzpatrick D (2000). Seeing beyond the receptive field in primary visual cortex. Curr Opin Neurobiol.

[CR33] Floyer-Lea A, Wylezinska M, Kincses T, Matthews PM (2006). Rapid modulation of GABA concentration in human sensorimotor cortex during motor learning. J Neurophysiol.

[CR34] Frangou P, Correia M, Kourtzi Z (2018). GABA, not BOLD, reveals dissociable learning-dependent plasticity mechanisms in the human brain. Elife.

[CR35] Frangou P, Emir UE, Karlaftis VM, Nettekoven C, Hinson EL, Larcombe S, Bridge H, Stagg CJ, Kourtzi Z (2019). Learning to optimize perceptual decisions through suppressive interactions in the human brain. Nat Commun.

[CR36] Govindaraju V, Young K, Maudsley AA (2000). Proton NMR chemical shifts and coupling constants for brain metabolites. NMR Biomed.

[CR37] Granger P, Bourdonneau M, Assemat O, Piotto M (2007). NMR chemical shift measurements revisited: high precision measurements. Concept Magn Reson A.

[CR38] Greenhouse I, Noah S, Maddock RJ, Ivry RB (2016). Individual differences in GABA content are reliable but are not uniform across the human cortex. Neuroimage.

[CR39] Hammett ST, Cook E, Hassan O, Hughes CA, Rooslien H, Tizkar R, Larsson J (2020). GABA, noise and gain in human visual cortex. Neurosci Lett.

[CR40] Harauzov A, Spolidoro M, DiCristo G, De Pasquale R, Cancedda L, Pizzorusso T, Viegi A, Berardi N, Maffei L (2010). Reducing intracortical inhibition in the adult visual cortex promotes ocular dominance plasticity. J Neurosci.

[CR41] Harris AD, Puts NAJ, Barker PB, Edden RAE (2015). Spectral-editing measurements of GABA in the human brain with and without macromolecule suppression. Magn Reson Med.

[CR42] Huber E, Chang K, Alvarez I, Hundle A, Bridge H, Fine I (2019). Early blindness shapes cortical representations of auditory frequency within auditory cortex. J Neurosci.

[CR43] Ip IB, Berrington A, Hess AT, Parker AJ, Emir UE, Bridge H (2017). Combined fMRI-MRS acquires simultaneous glutamate and BOLD-fMRI signals in the human brain. Neuroimage.

[CR44] Ip IB, Emir UE, Parker AJ, Campbell J, Bridge H (2019). Comparison of neurochemical and BOLD signal contrast response functions in the human visual cortex. J Neurosci.

[CR45] Isaacson JS, Scanziani M (2011). How inhibition shapes cortical activity. Neuron.

[CR46] Just N (2020). Proton functional magnetic resonance spectroscopy in rodents. NMR Biomed.

[CR47] Kurcyus K, Annac E, Hanning NM, Harris AD, Oeltzschner G, Edden R, Riedl V (2018). Opposite dynamics of GABA and glutamate levels in the occipital cortex during visual processing. J Neurosci.

[CR48] Lane C, Kanjlia S, Omaki A, Bedny M (2015). "Visual" cortex of congenitally blind adults responds to syntactic movement. J Neurosci.

[CR49] Letzkus JJ, Wolff SBE, Luthi A (2015). Disinhibition, a circuit mechanism for associative learning and memory. Neuron.

[CR50] Levelt W (1965). On binocular rivalry.

[CR51] Lin Y, Stephenson MC, Xin L, Napolitano A, Morris PG (2012). Investigating the metabolic changes due to visual stimulation using functional proton magnetic resonance spectroscopy at 7 T. J Cereb Blood Flow Metab.

[CR52] Lin AD, Andronesi O, Bogner W, Choi IY, Coello E, Cudalbu C, Juchem C, Kemp GJ, Kreis R, Krssak M, Lee P, Maudsley AA, Meyerspeer M, Mlynarik V, Near J, Oz G, Peek AL, Puts NA, Ratai EM, Tkac I, Mullins PG, Sta EWGR (2021). Minimum reporting standards for in vivo magnetic resonance spectroscopy (MRSinMRS): experts' consensus recommendations. NMR Biomed.

[CR53] Lunghi C, Burr DC, Morrone MC (2013). Long-term effects of monocular deprivation revealed with binocular rivalry gratings modulated in luminance and in color. J Vis.

[CR54] Lunghi C, Emir UE, Morrone MC, Bridge H (2015). Short-term monocular deprivation alters GABA in the adult human visual cortex. Curr Biol CB.

[CR55] Lynn J, Woodcock EA, Anand C, Khatib D, Stanley JA (2018). Differences in steady-state glutamate levels and variability between 'non-task-active' conditions: evidence from H-1 fMRS of the prefrontal cortex. Neuroimage.

[CR56] Mangia S, Tkac I, Gruetter R, Van De Moortele PF, Giove F, Maraviglia B, Ugurbil K (2006). Sensitivity of single-voxel 1H-MRS in investigating the metabolism of the activated human visual cortex at 7 T. Magn Reson Imaging.

[CR57] Mangia S, Tkac I, Logothetis NK, Gruetter R, Van de Moortele PF, Ugurbil K (2007). Dynamics of lactate concentration and blood oxygen level-dependent effect in the human visual cortex during repeated identical stimuli. J Neurosci Res.

[CR58] Maya Vetencourt JF, Sale A, Viegi A, Baroncelli L, De Pasquale R, O'Leary OF, Castren E, Maffei L (2008). The antidepressant fluoxetine restores plasticity in the adult visual cortex. Science.

[CR59] Mekle R, Kuhn S, Pfeiffer H, Aydin S, Schubert F, Ittermann B (2017). Detection of metabolite changes in response to a varying visual stimulation paradigm using short-TE H-1 MRS at 7 T. Nmr Biomed.

[CR60] Mentch J, Spiegel A, Ricciardi C, Robertson CE (2019). GABAergic inhibition gates perceptual awareness during binocular rivalry. J Neurosci.

[CR61] Mescher M, Merkle H, Kirsch J, Garwood M, Gruetter R (1998). Simultaneous in vivo spectral editing and water suppression. NMR Biomed.

[CR62] Mikkelsen M, Harris AD, Edden RAE, Puts NAJ (2018). Macromolecule-suppressed GABA measurements correlate more strongly with behavior than macromolecule-contaminated GABA plus measurements. Brain Res.

[CR63] Mikkelsen M, Loo RS, Puts NAJ, Edden RAE, Harris AD (2018). Designing GABA-edited magnetic resonance spectroscopy studies: considerations of scan duration, signal-to-noise ratio and sample size. J Neurosci Meth.

[CR64] Mullins PG (2018). Towards a theory of functional magnetic resonance spectroscopy (fMRS): a meta-analysis and discussion of using MRS to measure changes in neurotransmitters in real time. Scand J Psychol.

[CR65] Mullins PG, McGonigle DJ, O'Gorman RL, Puts NA, Vidyasagar R, Evans CJ, Symposium C (2014). Current practice in the use of MEGA-PRESS spectroscopy for the detection of GABA. Neuroimage.

[CR66] Muthukumaraswamy SD, Edden RAE, Jones DK, Swettenham JB, Singh KD (2009). Resting GABA concentration predicts peak gamma frequency and fMRI amplitude in response to visual stimulation in humans. P Natl Acad Sci USA.

[CR67] Near J, Harris AD, Juchem C, Kreis R, Marjanska M, Oz G, Slotboom J, Wilson M, Gasparovic C (2020). Preprocessing, analysis and quantification in single-voxel magnetic resonance spectroscopy: experts' consensus recommendations. NMR Biomed.

[CR68] Ooi TL, He ZJ (2020). Sensory eye dominance: relationship between eye and brain. Eye Brain.

[CR69] Park HJ, Lee JD, Kim EY, Park B, Oh MK, Lee S, Kim JJ (2009). Morphological alterations in the congenital blind based on the analysis of cortical thickness and surface area. Neuroimage.

[CR70] Pitchaimuthu K, Wu QZ, Carter O, Nguyen BN, Ahn S, Egan GF, McKendrick AM (2017). Occipital GABA levels in older adults and their relationship to visual perceptual suppression. Sci Rep.

[CR71] Posse S, Otazo R, Dager SR, Alger J (2013). MR spectroscopic imaging: principles and recent advances. J Magn Reson Imaging.

[CR72] Prichard J, Rothman D, Novotny E, Petroff O, Kuwabara T, Avison M, Howseman A, Hanstock C, Shulman R (1991). Lactate rise detected by 1H NMR in human visual cortex during physiologic stimulation. Proc Natl Acad Sci U S A.

[CR73] Puts NAJ, Edden RAE (2012). In vivo magnetic resonance spectroscopy of GABA: a methodological review. Prog Nucl Mag Res Sp.

[CR74] Rae CD (2014). A guide to the metabolic pathways and function of metabolites observed in human brain H-1 magnetic resonance spectra. Neurochem Res.

[CR75] Reislev NL, Dyrby TB, Siebner HR, Kupers R, Ptito M (2016). Simultaneous assessment of white matter changes in microstructure and connectedness in the blind brain. Neural Plast.

[CR76] Rideaux R (2020). Temporal dynamics of GABA and Glx in the visual cortex. Eneuro.

[CR77] Rideaux R, Goncalves NR, Welchman AE (2019). Mixed-polarity random-dot stereograms alter GABA and Glx concentration in the early visual cortex. J Neurophysiol.

[CR78] Robertson EM, Pascual-Leone A, Miall RC (2004). Current concepts in procedural consolidation. Nat Rev Neurosci.

[CR79] Robertson CE, Ratai EM, Kanwisher N (2016). Reduced GABAergic action in the autistic brain. Curr Biol CB.

[CR80] Rothman DL, Behar KL, Prichard JW, Petroff OAC (1997). Homocarnosine and the measurement of neuronal pH in patients with epilepsy. Magnet Reson Med.

[CR81] Sandberg K, Blicher JU, Del Pin SH, Andersen LM, Rees G, Kanai R (2016). Improved estimates for the role of grey matter volume and GABA in bistable perception. Cortex.

[CR82] Schaller B, Mekle R, Xin L, Kunz N, Gruetter R (2013). Net increase of lactate and glutamate concentration in activated human visual cortex detected with magnetic resonance spectroscopy at 7 tesla. J Neurosci Res.

[CR83] Schallmo MP, Kale AM, Millin R, Flevaris AV, Brkanac Z, Edden RA, Bernier RA, Murray SO (2018). Suppression and facilitation of human neural responses. Elife.

[CR84] Schallmo MP, Millin R, Kale AM, Kolodny T, Edden RAE, Bernier RA, Murray SO (2019). Glutamatergic facilitation of neural responses in MT enhances motion perception in humans. Neuroimage.

[CR85] Schallmo MP, Kolodny T, Kale AM, Millin R, Flevaris AV, Edden RAE, Gerdts J, Bernier RA, Murray SO (2020). Weaker neural suppression in autism. Nat Commun.

[CR86] Seely J, Chow CC (2011). Role of mutual inhibition in binocular rivalry. J Neurophysiol.

[CR87] Sengpiel F, Jirmann KU, Vorobyov V, Eysel UT (2006). Strabismic suppression is mediated by inhibitory interactions in the primary visual cortex. Cereb Cortex.

[CR88] Shapley R, Hawken M, Ringach DL (2003). Dynamic's of orientation selectivity in the primary visual cortex and the importance of cortical inhibition. Neuron.

[CR89] Shen J, Petersen KF, Behar KL, Brown P, Nixon TW, Mason GF, Petroff OAC, Shulman GI, Shulman RG, Rothman DL (1999). Determination of the rate of the glutamate glutamine cycle in the human brain by in vivo C-13 NMR. P Natl Acad Sci USA.

[CR90] Shibata K, Sasaki Y, Bang JW, Walsh EG, Machizawa MG, Tamaki M, Chang LH, Watanabe T (2017). Overlearning hyperstabilizes a skill by rapidly making neurochemical processing inhibitory-dominant. Nat Neurosci.

[CR91] Staermose TG, Knudsen MK, Kasch H, Blicher JU (2019). Cortical GABA in migraine with aura—an ultrashort echo magnetic resonance spectroscopy study. J Headache Pain.

[CR92] Stanley JA, Raz N (2018). Functional magnetic resonance spectroscopy: the "New" MRS for cognitive neuroscience and psychiatry research. Front Psychiatry.

[CR93] Steel A, Chiew M, Jezzard P, Voets NL, Plaha P, Thomas MA, Stagg CJ, Emir UE (2018). Metabolite-cycled density-weighted concentric rings k-space trajectory (DW-CRT) enables high-resolution 1 H magnetic resonance spectroscopic imaging at 3-Tesla. Sci Rep.

[CR94] Tadin D, Lappin JS, Gilroy LA, Blake R (2003). Perceptual consequences of centre-surround antagonism in visual motion processing. Nature.

[CR95] Tadin D, Silvanto J, Pascual-Leone A, Battelli L (2011). Improved motion perception and impaired spatial suppression following disruption of cortical area MT/V5. J Neurosci.

[CR96] Tkac I, Andersen P, Adriany G, Merkle H, Ugurbil K, Gruetter R (2001). In vivo H-1 NMR spectroscopy of the human brain at 7 T. Magnet Reson Med.

[CR97] Tkac I, Oz G, Adriany G, Ugurbil K, Gruetter R (2009). In vivo H-1 NMR spectroscopy of the human brain at high magnetic fields: metabolite quantification at 4T vs. 7T. Magnet Reson Med.

[CR98] van Loon AM, Knapen T, Scholte HS, St John-Saaltink E, Donner TH, Lamme VA (2013). GABA shapes the dynamics of bistable perception. Curr Biol CB.

[CR99] Watkins KE, Cowey A, Alexander I, Filippini N, Kennedy JM, Smith SM, Ragge N, Bridge H (2012). Language networks in anophthalmia: maintained hierarchy of processing in 'visual' cortex. Brain.

[CR100] Watkins KE, Shakespeare TJ, O'Donoghue MC, Alexander I, Ragge N, Cowey A, Bridge H (2013). Early auditory processing in area V5/MT+ of the congenitally blind brain. J Neurosci.

[CR101] Weaver KE, Richards TL, Saenz M, Petropoulos H, Fine I (2013). Neurochemical changes within human early blind occipital cortex. Neuroscience.

[CR102] Wilson M, Andronesi O, Barker PB, Bartha R, Bizzi A, Bolan PJ, Brindle KM, Choi IY, Cudalbu C, Dydak U, Emir UE, Gonzalez RG, Gruber S, Gruetter R, Gupta RK, Heerschap A, Henning A, Hetherington HP, Huppi PS, Hurd RE, Kantarci K, Kauppinen RA, Klomp DWJ, Kreis R, Kruiskamp MJ, Leach MO, Lin AP, Luijten PR, Marjanska M, Maudsley AA, Meyerhoff DJ, Mountford CE, Mullins PG, Murdoch JB, Nelson SJ, Noeske R, Oz G, Pan JW, Peet AC, Poptani H, Posse S, Ratai EM, Salibi N, Scheenen TWJ, Smith ICP, Soher BJ, Tkac I, Vigneron DB, Howe FA (2019). Methodological consensus on clinical proton MRS of the brain: review and recommendations. Magnet Reson Med.

[CR103] Wu L, Tang Z, Sun X, Feng X, Qian W, Wang J, Jin L (2013). Metabolic changes in the visual cortex of binocular blindness macaque monkeys: a proton magnetic resonance spectroscopy study. PLoS ONE.

[CR104] Yoon JH, Maddock RJ, Rokem A, Silver MA, Minzenberg MJ, Ragland JD, Carter CS (2010). GABA concentration is reduced in visual cortex in schizophrenia and correlates with orientation-specific surround suppression. J Neurosci.

